# Relationship between subjective cognitive concerns and objective neurocognitive performance in primary medical versus primary psychiatric populations

**DOI:** 10.1017/S0033291725001084

**Published:** 2025-05-22

**Authors:** Matthew S. Phillips, George Whitman Kent, Hajar Ismail, Justyna Piszczor, Briana N. Galindo, Greg Shapiro, Lisette Gonzalez, Brian M. Cerny, Jason R. Soble

**Affiliations:** 1 Department of Psychiatry, University of Illinois College of Medicine, Chicago, IL, USA; 2 Department of Psychology, Wheaton College, Wheaton, IL, USA; 3 Department of Psychology, Roosevelt University, Chicago, IL, USA; 4 Department of Clinical Psychology, The Chicago School, Chicago, IL, USA; 5 Department of Psychology, University of Illinois at Chicago, Chicago, IL, USA; 6 Department of Neurology, University of Illinois College of Medicine, Chicago, IL, USA

**Keywords:** anxiety, assessment, cognition, depression, neuropsychology, subjective cognitive concerns

## Abstract

**Background:**

Subjective cognitive concerns (SCCs) refer to individuals’ self-identified cognitive limitations, irrespective of objective neurocognitive performance. Previous literature has overwhelmingly found that psychiatric factors, not neurocognitive dysfunction, are a primary correlate of elevated SCCs across a wide range of clinical populations. However, the relationship between SCCs and objective neurocognitive performance is complex and may further be influenced by underlying mechanisms of various impairments or etiologies. Moreover, much of the extant literature has under-utilized performance validity tests (PVTs) when analyzing objective neuropsychological outcomes.

**Methods:**

As such, this study examined the associations between SCCs, performance validity, neurocognitive performance, and psychiatric distress among adult clinical patients with primary medical/neurologic (n = 127) and psychiatric (n = 106) etiologies.

**Results:**

Results showed that elevated SCCs are associated with greater degrees of performance invalidity and psychiatric distress, but not neurocognitive performance, among both groups.

**Conclusions:**

Findings support the utility of PVTs in clinical research and further highlight the impact of psychiatric factors on SCCs, regardless of medical/neurologic or psychiatric etiology.

## Introduction

Subjective cognitive concerns (SCCs) pertain to individuals’ self-identified worries regarding their cognitive capabilities, irrespective of objective neurocognitive assessment outcomes (Jessen et al., 2020; Mendonça, Alves, & Bugalho, 2016). Historically, research has found that the prevalence of SCCs generally tends to increase with age-related cognitive decline and/or comorbid medical conditions such as Alzheimer’s disease, vascular dementia, and Parkinson’s syndromes (Mitchell, 2008). Among these populations, studies have identified stronger associations between increased SCCs and objective neurocognitive decline (Barbosa et al., 2019; Dufouil et al., 2005; Hong & Lee, 2023; Mitchell et al., 2014), although this finding certainly is not ubiquitous. Notably, anosognosia associated with Alzheimer’s disease often leads to unique challenges as patients tend to be rather oblivious of their objective decline, especially as the disease progresses (Cacciamani et al., 2021). Other studies have demonstrated that SCCs have no relationship with objective metrics of cognitive impairment and result in overestimation of cognitive problems and higher rates of mild cognitive impairment misdiagnosis (Edmonds et al., 2014). More recently, research has begun to explore the relationship between SCCs and objective cognitive functioning in other clinical populations.

Indeed, although SCCs often prompt a referral for neuropsychological evaluation, other factors – aside from frank neurocognitive dysfunction may be driving these concerns. In a large systematic review, Burmester, Leathem, and Merrick (2016) found that while small but significant associations were identified between SCCs and objective neurocognitive performance across a large number of studies/age groups, psychiatric factors (i.e. depressive symptoms) were the primary contributor. This same review also identified a number of methodological limitations in many extant studies that likely contributed to the small association that was found between SCCs and objective findings, including the use of brief cognitive screeners and unvalidated self-report questionnaires, inclusion of volunteer samples not representative of clinical populations, and failure to include any measures of affective symptomatology.

When analyzing SCCs among broader clinical populations, similar findings have emerged. For instance, among individuals with mild traumatic brain injury (mTBI), Donnelly et al. (2018) reported that SCCs did not correspond with objective cognitive functioning and were more strongly associated with psychiatric distress. This is unsurprising given that post-concussion symptom complaints are nonspecific to mTBI and are associated with active psychopathology (Donnell, Kim, Silva, & Vanderploeg, 2012; Soble et al., 2014). Moreover, Finley et al. (2024) found that cognitive performance had a weak correlation with SCCs but strong associations with internalizing psychopathology and somatic symptoms in a sample of non-geriatric adults with confirmed ADHD. Similarly, in primary neurological populations (i.e. epilepsy, head injury, and cancer), increased depression and psychosocial stress, but not objective neurocognitive dysfunction, are highly correlated with an increase in SCCs (Galioto et al., 2015; Pullens et al., 2010; Van Patten et al., 2024). Serra-Blasco et al. (2019) found that patients with primary psychiatric diagnoses experiencing acute depressive episodes were more likely to underestimate their cognitive abilities on measures of attention and memory, suggesting that specific cognitive domains may be more vulnerable to SCCs.

To complicate matters, one major limitation of many previous studies exploring SCCs is the omission of performance validity tests (PVTs) to ensure valid objective cognitive test data. Although the literature in this area is sparse, a few select studies have explored the impact of PVTs on SCCs and objective neurocognitive functioning. Among general memory clinic patients, Czornik, Merten, and Lehrner (2021) examined PVTs in patients with SCCs and found that only about 7% of their patients invalidated neurocognitive tests. These findings align more closely with previous literature identifying a positive association between SCCs and objective neurocognitive deficits in older adult populations (Barbosa et al., 2019; Dufouil et al., 2005; Hong & Lee, 2023; Mitchell et al., 2014). Conversely, Phillips et al. (2025) found that, among young adults referred for ADHD evaluation, those who reported elevated SCCs were almost twice as likely to invalidate PVTs as those who did not, thus calling into question the validity of SCCs in younger, relatively healthier samples.

Overall, the relationship between SCCs and objective cognitive performance is complex and may be further influenced by the underlying mechanisms of various impairments or etiologies. Theoretically, medical/neurologic and psychiatric conditions have distinct associations with cognitive functioning (Vance et al., 2016). For instance, while some medical conditions may lead to cognitive deficits through direct physiological damage or systemic effects (Gonzales et al., 2022), psychiatric disorders often distort cognitive perceptions through emotional and psychological processes, including perceptions of one’s own cognitive performance (Groenman, van der Werf, & Geurts, 2022). This divergence raises important questions about how different types of etiologies influence the relationship between SCCs and objective neurocognitive functioning/impairment. To address this question, this study aimed to build on existing literature by examining whether the association between SCCs and objective neurocognitive performance differs based on the primary etiology of cognitive complaints and/or dysfunction. Specifically, the study dichotomized neuropsychological examinees by primary psychiatric and medical/neurologic conditions and compared objective neurocognitive performance between those with and without significant SCCs after controlling for performance validity using a series of well-validated PVTs. Based on previous literature, it was hypothesized that SCCs would be more strongly influenced by psychiatric factors (i.e. depression and anxiety) in both psychiatric and medical/neurologic groups, with a stronger relationship in primary psychiatric patients. It was also hypothesized that individuals with elevated SCCs would be more likely to invalidate PVTs compared to those with unelevated SCCs.

## Method

### Participants & procedures

Cross-sectional data from 393 adult clinical patients referred for comprehensive outpatient neuropsychological evaluation at an urban academic medical center were analyzed. All patients provided written consent for their data to be used for research purposes as part of an ongoing university-based IRB-approved study. Patients’ neuropsychological diagnostic workup included an extensive medical record review, a comprehensive history questionnaire, a clinical interview, a neuropsychological battery to characterize cognitive status, a series of face-valid self-report measures of mood/anxiety, and the Minnesota Multiphasic Personality Inventory-2-Restructured Form (MMPI-2-RF; Ben-Porath & Tellegen, 2008). One hundred twenty-six patients were not administered the MMPI-2-RF, most often due to significant cognitive impairment, which precluded their ability to tolerate the protocol, or, in a minority of cases, due to time limitations, resulting in their exclusion. An additional 25 patients had evidence of excessive variable or fixed, content-inconsistent responding on the MMPI-2-RF (i.e. VRIN-r and/or TRIN-r ≥ 80 T) and were also excluded. Finally, nine patients received no diagnosis or did not receive a primary medical or psychiatric diagnosis, which were the conditions of interest for this study, and were excluded. This resulted in a final study sample of 233 diverse adult outpatients (see Table 1).

**Table 1.** Demographics for the total sample

	Non-elevated COG (*n* = 78)		Elevated COG (*n* = 155)			
	*M* (*SD*)	Range	*M* (*SD*)	Range	*F*	*η_p_^2^*
Age	43.91 (14.13)	19–73	39.84 (14.09)	18–69	4.326*	.018
Education	14.67 (2.58)	8–20	14.16 (2.59)	7–20	1.977	.008
Predicted FSIQ	99.73 (10.97)	72–128	97.42 (11.38)	68–123	2.192	.009
	*N*	%	*N*	%	*X^2^*	Cramer’s V
Sex					0.103	.749
Male	32	41%	67	43%		
Female	46	59%	88	57%		
Race/Ethnicity					2.464	.651
White	35	45%	62	40%		
Black	11	14%	32	21%		
Hispanic	27	35%	50	32%		
Asian American	3	4%	9	6%		
Other	2	2%	2	1%		
PVT performance					8.58**	.192
Valid (0–1 failures)	71	91%	116	75%		
Invalid (≥2 failures)	7	9%	39	25%		

*Note:* * *p* < .05; ** < .01; PVT, Performance Validity Test; COG, Minnesota Multiphasic Personality Inventory-2-Restructured Form Cognitive Complaints scale.

### Measures

#### Neuropsychological test battery

The neuropsychological test battery included all of the following measures: Rey Auditory Verbal Learning Test (RAVLT; Schmidt, 1996), Weschler Adult Intelligence Scale-Fourth Edition (WAIS-IV) Processing Speed (PSI) index and Digit Span (Wechsler, 2008), Trail Making Test A and B (TMT-A; TMT-B; Heaton, 2004), Stroop Color and Word Test-Inhibition Trial (SCWT; Golden, 1978), and Letter (FAS) and Semantic (Animal Naming) Verbal Fluency Tests (Heaton, 2004). The battery also contained four well-validated, freestanding PVTs: Dot Counting Test (Boone, Lu, & Herzberg, 2002), Medical Symptom Validity Test (Green, 2004; Resch, Rhoads, Ovsiew, & Soble, 2022), Test of Memory Malingering-Trial 1 (Martin et al., 2020), and Word-Choice Test (Bernstein, Resch, Ovsiew, & Soble, 2021). For analyses examining performance validity status, patients with 0–1 failures across the four freestanding PVTs were classified as having valid neuropsychological test performance, and those with ≥2 failures were classified as having invalid test performance (Jennette et al., 2021; Martin, Schroeder, & Olsen, 2022; Sweet et al., 2021).

#### *Minnesota multiphasic personality inventory-2-restructured form (MMPI-2-RF;* Ben-Porath & Tellegen, *2008)*

The MMPI-2-RF is an objective measure of psychiatric symptoms and personality features, consisting of 338 true/false questions. It contains 10 validity scales that are used to assess inconsistent responding and over- and underreporting, as well as nine clinical scales and 30 specific problem scales. T-scores ≥65 suggest elevations across the clinical and specific problems scales. The main scale of interest for this study was the Cognitive Complaints (COG) scale, which provides insight into one’s subjective report of cognitive difficulties involving concentration, memory, intelligence challenges, and general confusion. As noted above, patients with invalid VRIN-r and/or TRIN-r validity scores were entirely excluded from the study. For the remaining validity scales, definite overreporting was operationalized as ≥120 T on F-r, ≥100 on Fp-r, Fs, FBR-r, and RBS, and definite underreporting as ≥80 T on L-r and ≥ 70 on K-r (Ben-Porath, 2012).

#### ***Beck** depression inventory-2*nd *edition* (BDI-II; Beck, Steer, & Brown, *1996) and beck anxiety inventory* (BAI; Beck, Epstein, Brown, & Steer, 1988).

Both the BDI-II and BAI are face-valid self-report measures of depression and anxiety symptoms, respectively, consisting of 21 items (scores range from 0–63), with higher scores indicating more severe symptomatology.

#### Data analysis

Data were analyzed using IBM SPSS statistics software (Version 27; IBM Corp, 2020). All statistical assumptions were met before running analyses (e.g. test of normality and multicollinearity). The overall sample was dichotomized to form primary medical/neurologic and primary psychiatric etiology groups (Medical/Neurologic = 127; Psychiatric = 106) based on whether the referral was based on a primary medical/neurologic problem or a psychiatric problem (see Table 2 for breakdown of subgroup etiologies). The MMPI-2-RF COG scale was dichotomized into elevated (≥65 T) or non-elevated (≤64 T) to create groups based on scale elevations for the entire sample, as well as for each individual study sample (see Tables 3 and 4). The four freestanding PVTs discussed above were used to form valid (*n* = 187) and invalid (*n* = 46) neurocognitive performance groups. Supplementary analyses were also conducted on a subsample that showed evidence of no-definite overreporting or underreporting on the MMPI-2-RF (*n* = 178).

**Table 2.** Specific diagnoses by study group

Primary medical/neurologic etiology diagnoses	*n*
Schizophrenia spectrum	8
Cerebrovascular disease/insult	15
Amnestic MCI	2
Multiple sclerosis/autoimmune	10
Alcohol/substance-induced cognitive decline	5
TBI	21
Seizure/epilepsy	11
Tumor/neoplasm	7
Non-CNS cancer/chemotherapy	3
HIV	2
Aneurysm/AVM	5
Sleep apnea	3
COVID–19	3
Electrical injury	1
Multiple etiologies	11
Other	20
Primary psychiatric etiology diagnoses	*n*
PTSD	11
Depression	19
Bipolar disorder	3
Personality disorder	2
Anxiety	7
ADHD	27
Learning disorder	4
Substance use disorder	3
Somatic symptom disorder	6
Insomnia	4
Pain	11
Borderline intellectual functioning	1
Gender dysphoria	1
Multifactorial/other	7

*Note:* MCI, Mild Cognitive Impairment; TBI, Traumatic Brain Injury; HIV, Human Immunodeficiency Virus; AVM, Arteriovenous Malformation; PTSD, Posttraumatic Stress Disorder; ADHD, Attention-Deficit/Hyperactivity Disorder.

**Table 3.** Validity status based on primary etiology

	Non-elevated COG		Elevated COG			
Primary medical/neurologic PVT performance	*N* = 46	%	*N* = 81	%	X^2^	Fisher’s exact
					2.42	.166
Valid	43	93%	68	84%		
Invalid	3	7%	13	16%		
Primary psychiatric PVT Performance	*N* = 32	%	*N* = 74	%	5.64	.019
Valid	28	88%	48	65%		
Invalid	4	12%	26	35%		

*Note:* PVT, Performance Validity Test; COG, Minnesota Multiphasic Personality Inventory-2-Restructured Form Cognitive Complaints scale.

**Table 4.** Validity status based on primary etiology for no-definite MMPI-2-RF invalidity group

	Non-elevated COG		Elevated COG			
Primary medical/neurologic PVT performance	*N* = 39	%	*N* = 53	%	X^2^	Fisher’s exact
					.705	.509
Valid	36	92%	46	87%		
Invalid	3	8%	7	13%		
Primary psychiatric PVT Performance	*N* = 30	%	*N* = 56	%	2.77	.154
Valid	27	90%	42	75%		
Invalid	3	10%	14	25%		

*Note:* PVT, Performance Validity Test; COG, Minnesota Multiphasic Personality Inventory-2-Restructured Form Cognitive Complaints scale.

Chi-square tests were used to assess for differences in performance validity status (i.e. valid or invalid) based on elevated and non-elevated COG scores for the entire sample, as well as the primary medical/neurologic and psychiatric etiology groups. To determine if evidence of overreporting on the MMPI-2-RF influenced this relationship, an additional chi-square was conducted to assess differences in the subsample of patients with no-definite overreporting or underreporting. Analyses of variance (ANOVAs) were conducted to test for significant differences in objective neurocognitive performance between the elevated and non-elevated COG groups among those with primary medical/neurologic and primary psychiatric etiologies. To correct for the familywise error rate due to multiple comparisons, the false discovery rate (FDR) procedure was applied with a .05 maximum FDR (Benjamini & Hochberg, 1995). ANOVAs were repeated among the primary etiology groups in the no-definite MMPI-2-RF overreporting or underreporting subsample with FDR corrections. Finally, a series of linear regressions assessed if depression/distress and anxiety using the BDI-II, MMPI-2-RF RCd scale, and BAI predicted COG score elevations between the primary medical and primary psychiatric etiology groups.

## Results

Demographic characteristics for the elevated and non-elevated COG groups are in Table 1. In the overall sample, the elevated COG group had higher rates of invalid neuropsychological test performance based on PVT failures compared to the non-elevated COG group. A similar pattern was observed in the primary medical/neurologic and primary psychiatric groups, such that those in the primary psychiatric etiology group with elevated COG scores showed about twice the rate of invalid PVT performance than the primary medical/neurologic etiology group (Table 3). Among the no-definite MMPI-2-RF overreporting group, again a similar pattern emerged wherein elevated COG groups showed a greater degree of PVT failure with the primary psychiatric etiology group showing a higher rate of PVT failure than the primary medical/neurologic etiology group (Table 4).

ANOVAs assessing differences in neuropsychological test performance between elevated and non-elevated COG scores among the primary medical/neurologic and primary psychiatric etiology groups are presented in Table 5. In short, no significant differences were observed for either etiology group after controlling for the familywise error rate. After excluding those with evidence of MMPI-2-RF overreporting (Table 6), a similar pattern emerged.

**Table 5.** All MMPI-2-RF group

Primary medical/neurologic	Non-elevated COG (*n* = 46)	Elevated COG (*n* = 81)			
Cognitive test	*M* (*SD*)	*M* (*SD*)	*F*	*p*	*η_p_^2^*
RAVLT trials 1–5	37.39 (14.98)	36.21 (11.00)	0.259	.814	.002
RAVLT delay trial	39.28 (13.89)	38.32 (12.06)	0.167	.814	.001
WAIS-IV PSI	94.98 (12.17)	89.41 (13.51)	5.353	.176	.041
WAIS-IV digit span	8.96 (2.69)	8.26 (2.60)	2.060	.913	.016
Trails A	46.85 (11.63)	46.62 (11.30)	0.012	.814	.000
Trails B	44.13 (12.35)	42.53 (12.00)	0.462	.814	.004
Stroop color-word	49.67 (7.90)	51.48 (10.01)	1.108	.814	.009
Letter fluency	45.48 (11.88)	44.23 (11.60)	0.332	.814	.003
Category fluency	45.15 (10.63)	44.35 (12.45)	0.136	.814	.001
Primary psychiatric	Non-elevated COG (*n* = 32)	Elevated COG (*n* = 73)			
Cognitive test	*M* (*SD*)	*M* (*SD*)	*F*	*p*	*η_p_^2^*
RAVLT trials 1–5	44.44 (11.92)	40.15 (12.58)	2.667	.236	.025
RAVLT delay trial	44.56 (14.48)	42.82 (12.30)	0.400	.595	.004
WAIS-IV PSI	103.56 (16.81)	94.52 (16.33)	6.699	.099	.061
WAIS-IV digit span	9.34 (3.00)	8.81 (2.98)	0.715	.514	.007
Trails A	53.81 (14.78)	47.95 (14.21)	3.702	.236	.035
Trails B	46.22 (11.36)	45.40 (12.29)	0.104	.748	.001
Stroop color-word	52.87 (8.43)	50.44 (7.80)	2.068	.269	.020
Letter fluency	48.72 (12.26)	45.44 (11.06)	1.831	.269	.017
Category fluency	47.94 (9.51)	44.14 (11.40)	2.722	.236	.026

*Note:* There were no statistically significant findings across cognitive tests. RAVLT, Rey Auditory Verbal Learning Test; WAIS-IV, Wechsler Adult Intelligence Test, 4th Edition; PSI, Processing Speed Index; Trails, Trail Making Test; COG, Minnesota Multiphasic Personality Inventory-2-Restructured Form Cognitive Complaints. All *p*-values reflect false discovery rate-corrected *p*-values.

**Table 6.** No-Definite MMPI-2-RF Invalidity

Primary Medical/Neurologic	Non-Elevated COG (*n* = 39)	Elevated COG (*n* = 53)			
Cognitive Test	*M* (*SD*)	*M* (*SD*)	*F*	*p*	*η_p_^2^*
RAVLT Trials 1–5	38.69 (14.33)	38.47 (10.34)	0.007	.973	.000
RAVLT Delay Trial	38.95 (14.25)	40.70 (11.65)	0.419	.973	.005
WAIS-IV PSI	95.77 (12.22)	90.28 (13.67)	3.954	.450	.042
WAIS-IV Digit Span	9.13 (2.79)	8.38 (2.90)	1.551	.972	.017
Trails A	47.85 (11.53)	47.30 (9.91)	0.059	.973	.001
Trails B	44.15 (12.90)	43.02 (13.33)	0.167	.973	.002
Stroop Color-Word	49.62 (8.09)	49.55 (10.38)	0.001	.973	.000
Letter Fluency	46.26 (12.12)	46.04 (11.75)	0.008	.973	.000
Category Fluency	45.51 (10.81)	45.09 (12.17)	0.029	.973	.000
Primary Psychiatric	Non-Elevated COG (*n* = 30)	Elevated COG (*n* = 56)			
Cognitive Test	*M* (*SD*)	*M* (*SD*)	*F*	*p*	*η_p_^2^*
RAVLT Trials 1–5	45.37 (11.73)	41.77 (12.72)	1.650	.203	.019
RAVLT Delay Trial	45.33 (14.44)	44.14 (11.61)	0.173	.679	.002
WAIS-IV PSI	105.83 (14.67)	96.34 (16.47)	6.990	.090	.077
WAIS-IV Digit Span	9.60 (2.90)	8.75 (3.08)	1.548	.279	.018
Trails A	54.27 (15.17)	49.14 (13.75)	2.523	.209	.029
Trails B	47.90 (9.51)	45.71 (12.28)	0.718	.449	.008
Stroop Color-Word	53.67 (8.10)	50.36 (8.03)	3.302	.209	.038
Letter Fluency	49.17 (12.55)	44.98 (10.56)	2.687	.209	.031
Category Fluency	48.50 (8.98)	43.93 (11.09)	3.768	.209	.043

*Note.* There were no statistically significant findings across cognitive tests. RAVLT: Rey Auditory Verbal Learning Test; WAIS-IV: Wechsler Adult Intelligence Test, 4th Edition; PSI: Processing Speed Index; Trails: Trail Making Test; COG: Minnesota Multiphasic Personality Inventory-2-Restructured Form Cognitive Complaints. All *p*-values reflect false discovery rate-corrected *p*-values.

Finally, among the primary medical/neurologic etiology group, both depression/psychological distress (BDI-II, R^2^ = .203; *p* < .001; RCd, R^2^ = .364: *p* < .001) and anxiety (BAI, R^2^ = .139, *p* = <.001) symptoms significantly predicted SCCs whereby those with greater self-reported depression and anxiety also endorsed higher rates of SCCs. An additional regression that combined the BDI-II and BAI was also significant and found greater shared variance than the BDI-II and BAI models alone (R^2^ = .221, *p* = <.001). Similar findings were observed among the primary psychiatric etiology group for the BDI-II (R^2^ = .077, *p* = .004), RCd scale (R^2^ = .115, *p* < .001), and BAI (R^2^ = .137, *p* < .001), and a regression combining the BDI-II and BAI found greater variance than with each of the models alone (R^2^ = .143, *p* < .001).

## Discussion

### Primary findings

The present study investigated the associations between subjective cognitive complaints (SCCs) and performance invalidity, cognitive performance, and psychiatric distress among clinical neuropsychological individuals stratified by medical/neurologic and psychiatric reasons for referral. Consistent with the primary hypothesis, results indicated that examinees with clinically elevated SCCs were more likely to produce invalid cognitive performance data, particularly those with primary psychiatric etiologies. Additionally, results also support the other primary hypothesis that, after accounting for performance validity, SCCs were unrelated to objective neurocognitive performance. Finally, psychiatric distress was significantly associated with SCCs among both groups, more so among those with medical/neurologic etiologies.

Higher rates of performance invalidity among those with elevated SCCs observed in the present study aligns with previous literature showing a similar relationship in those being referred for ADHD diagnostic evaluations (Phillips et al., *2025*). However, this current study also controlled for symptom invalidity to see how over- or underreporting might further drive this relationship. Notably, the relationship between PVT failure rates and SCC endorsement remained largely the same. To our knowledge, the relationship between SCCs and PVT failure rate has not been well explored apart from a few previous studies using distinct patient populations (Czornik et al., 2021; Nauta et al., 2022; *Phillips et al., 2025*). Overall, these findings significantly contribute to the current dearth of literature on the relationship between SCCs and PVT performance by suggesting that the use of PVTs in neuropsychological testing should be an integral component of the evaluation process.

The lack of differences between those with and without elevated SCCs observed in the present study support previous literature, which have found similar relationships in specific diagnostic samples (Finley et al., 2024; Ingulfsvann Hagen et al., 2023; Mulligan, Smart, & Ali, 2016; *Phillips et al., 2025*; Pranckeviciene, Deltuva, Tamasauskas, & Bunevicius, 2017; Serra-Blasco et al., 2019; Wahed et al., 2024; Zlatar et al., 2018), and extends results to a diagnostically diverse clinical sample. Therefore, it appears that a weak to null association between SCCs and objective cognitive performance is not limited to a circumscribed patient population. This finding strongly underscores the need for objective cognitive assessment among individuals with SCCs, as their cognitive symptom complaints are not indicative of actual cognitive dysfunction and are insufficient to warrant a neurocognitive disorder diagnosis. Primary care providers are, therefore, strongly encouraged to refer their patients with subjective cognitive complaints for comprehensive neuropsychological evaluation.

Also, similar to previous studies (Burmester et al., 2016; Edmonds et al., 2014; Finley et al., 2024; Pranckeviciene et al., 2017; Serra-Blasco et al., 2019; Topiwala et al., 2021), the present study observed a significant association between psychiatric symptoms and SCCs. SCCs were positively associated with depression and anxiety symptoms in both primary psychiatric groups and primary medical/neurologic groups. Shared variance was modest, indicating that SCCs are not related to, but not synonymous with, psychiatric distress. Contrary to hypotheses, a stronger association between SCCs and psychiatric distress was observed among the medical/neurologic group relative to the psychiatric group. Potential reasons include adjustment to medical/neurologic illness present in the medical/neurologic group and absent in the psychiatric group, leading to distorted appraisals of cognitive performance. It is also possible that items on the BDI-II commonly associated with medical/neurologic illnesses (e.g. restlessness/agitation, sleep dysfunction, fatigue, and changes in appetite) are responsible for the stronger association in the medical/neurologic group.

### Recommendations and future directions

Current study findings highlight the necessity of objective assessment of cognitive performance when patients present with SCCs. Further, cognitive assessments should include PVTs consistent with current practice standards (Sweet et al., 2021). Individuals with elevated SCCs have higher rates of performance invalidity, which represents a potential confound when investigating patients’ subjective appraisal of their cognitive abilities. This holds across both medical/neurologic and psychiatric contexts. Additionally, the present findings suggest that symptom validity testing alone is insufficient to disentangle SCCs from objective neurocognitive dysfunction. Even in those who did not over- or underreport on symptom validity tests (SVTs), those with elevated SCCs demonstrated a higher PVT failure rate. Taking these factors into account, findings suggest that researchers similarly utilize PVTs when assessing the relationship between SCCs and objective neuropsychological performance. Doing so will help increase the overall validity, reliability, and generalizability of future studies.

There remains debate as to whether SCCs may serve a role in predicting future decline, with some studies showing a significant relationship (Pike et al., 2022), others showing a stronger relationship with psychiatric factors (Edmonds et al., 2014; Hill et al., 2016; Topiwala et al., 2021), and those with mixed results (Brailean et al., 2019; Hill et al., 2016). Hill et al. (2016) even made a specific call for longitudinal research. However, at present, it remains clear that SCCs, objective neurocognitive functioning, and psychiatric distress are distinct constructs. Thus, future research using diverse clinical presentations should continue to explore potential correlates, predictors, and outcomes of SCCs.

### Strengths and limitations

This study has several strengths, including the use of a large and demographically diverse clinical sample representative of a general outpatient neuropsychological practice setting. Moreover, the present study utilized four well-validated criterion PVTs and a symptom validity-controlled self-report measure (i.e. the MMPI-2-RF) to establish the validity of test performance and self-reports among participants.

Despite the study’s strengths, it was not without limitations. For one, while the sample was diverse in many respects, participants were highly educated, and results may not generalize to patients with lower educational attainment. Furthermore, while the neurocognitive battery used in this study was comprehensive, it did not include some sub-domains often addressed in general clinical practice (e.g. visual and prose memory, naming, and visuospatial skills). In this respect, it is possible that the current battery of tests may not have been sensitive enough to capture nuanced differences across groups that other test measures may have identified. However, despite these limitations, the current battery was fairly extensive and included broad cognitive domains typically assessed during routine neuropsychological evaluations (e.g. attention/working memory, processing speed, memory, language, and executive functions).

## Conclusion

In sum, the results demonstrate that SCCs are a distinct construct from objective neurocognitive functioning and that SCCs are associated with psychiatric distress in both medical/neurologic and psychiatric populations. Additionally, this study further supports the need to include performance validity controls when assessing the credibility of patient’s subjective appraisal of neurocognitive functioning, even in those with valid symptom reporting.
